# Development of a Prognostic Model for Early Breast Cancer Integrating Neutrophil to Lymphocyte Ratio and Clinical-Pathological Characteristics

**DOI:** 10.1093/oncolo/oyad303

**Published:** 2023-11-16

**Authors:** Sara Socorro Faria, Diana Giannarelli, Vladmir Claudio Cordeiro de Lima, Sumadi Luckman Anwar, Chiara Casadei, Ugo De Giorgi, Gabriele Madonna, Paolo Antonio Ascierto, Rossana Veronica Mendoza López, Roger Chammas, Mariaelena Capone

**Affiliations:** Laboratory of Immunology and Inflammation, Department of Cell Biology, University of Brasilia, DF Brasilia, Brazil; Department of Epidemiology and Biostatistics, GSTeP, Fondazione Policlinico Universitario A. Gemelli, IRCCS, Rome, Italy; Department of Medical Oncology, A.C. Camargo Cancer Center, Sao Paulo, SPBrazil; Department of Surgery, Faculty of Medicine, Public Health and Nursing, Dr. Sardjito Hospital, Universitas Gadjah Mada, Yogyakarta, Indonesia; Department of Medical Oncology, IRCCS Instituto Romagnolo per lo Studio dei Tumori (IRST) “Dino Amadori,” Meldola, Italy; Department of Medical Oncology, IRCCS Instituto Romagnolo per lo Studio dei Tumori (IRST) “Dino Amadori,” Meldola, Italy; Department of Melanoma, Cancer Immunotherapy and Development Therapeutics. Istituto Nazionale Tumori IRCCS Fondazione “G. Pascale” Napoli, Italy; Department of Melanoma, Cancer Immunotherapy and Development Therapeutics. Istituto Nazionale Tumori IRCCS Fondazione “G. Pascale” Napoli, Italy; Center for Translational Research in Oncology, Institute of Cancer of São Paulo State, University of São Paulo, São Paulo, Brazil; Center for Translational Research in Oncology, Institute of Cancer of São Paulo State, University of São Paulo, São Paulo, Brazil; Department of Melanoma, Cancer Immunotherapy and Development Therapeutics. Istituto Nazionale Tumori IRCCS Fondazione “G. Pascale” Napoli, Italy

**Keywords:** breast cancer, external validation, neutrophil-to-lymphocyte ratio, prognostic model

## Abstract

**Background:**

Breast cancer-related inflammation is critical in tumorigenesis, cancer progression, and patient prognosis. Several inflammatory markers derived from peripheral blood cells count, such as the neutrophil-lymphocyte ratio (NLR), platelet-lymphocyte ratio (PLR), monocyte-lymphocyte ratio (MLR), and systemic immune-inflammation index (SII) are considered as prognostic markers in several types of malignancy.

**Methods:**

We investigate and validate a prognostic model in early patients with breast cancer to predict disease-free survival (DFS) based on readily available baseline clinicopathological prognostic factors and preoperative peripheral blood-derived indexes.

**Results:**

We analyzed a training cohort of 710 patients and 2 external validation cohorts of 980 and 157 patients with breast cancer, respectively, with different demographic origins. An elevated preoperative NLR is a better DFS predictor than others scores. The prognostic model generated in this study was able to classify patients into 3 groups with different risks of relapse based on ECOG-PS, presence of comorbidities, T and N stage, PgR status, and NLR.

**Conclusion:**

Prognostic models derived from the combination of clinicopathological features and peripheral blood indices, such as NLR, represent attractive markers mainly because they are easily detectable and applicable in daily clinical practice. More comprehensive prospective studies are needed to unveil their actual effectiveness.

## Teaser

This study aimed to establish and validate a prognostic model using an approach to predict disease-free survival based on readily available baseline clinical-pathological prognostic factors and preoperative blood count-derived lymphocyte ratios.

**Implications for Practice**

Neutrophil-to-lymphocyte ratio (NLR) has been identified as a prognostic marker for cancer risk stratification and therapy decision-making in many tumor types. However, it can be difficult to translate the NLR into a prognostic model of treatment due to the great variability in peripheral blood levels of leucocytes among different individuals. We developed and externally validated a prognostic model based on readily available clinical and hematological variables to estimate disease-free survival in early patients with breast cancer.

## Introduction

Breast cancer is the most common malignancy among women and is the second most prominent cause of cancer-related mortality worldwide.^1^ Breast cancer is a heterogeneous disease, encompassing different subtypes with distinct biological and clinical characteristics.^2,3^ Clinically, subtypes of breast cancer are defined by their histopathological appearance and expression of hormone receptors and growth factors (namely, the estrogen receptor (ER), the progesterone receptor (PgR), and the human epidermal growth factor receptor 2 (HER2).^4,5^ Traditional prognostic factors, such as tumor size, histological grade, vascular invasion, lymph node metastases, and distant metastases, have been routinely used to predict the outcomes.^6^ However, identifying novel prognostic factors might enable better risk stratification and treatment selection.^7^ It has become evident that breast cancer-related inflammation, both local and systemic, has an essential role in tumorigenesis, disease progression, and patient prognosis.^8^

Systemic inflammation is associated with alterations in peripheral blood leukocytes,^9^ and the neutrophil-to-lymphocyte ratio (NLR) has been identified as a prognostic indicator to refine risk stratification and guide therapy decisions in a variety of solid cancers.^10^ Neutrophils inhibit anti-tumor immunity by secreting many inflammatory mediators and suppressing the cytotoxic activity of T cells^11^; meanwhile, lymphocytes play a crucial role in immunosurveillance and immune editing, coordinating, and effecting the adaptive antitumoral immune response.^12^ However, little is known whether NLR is a surrogate for identifying a higher degree of neutrophils infiltration or correlates with a lower rate of lymphocytes in the tumor microenvironment.^13^

Inflammatory-related peripheral cells measured in routine blood tests (such as neutrophils, lymphocytes, and platelets) and their derived indexes, including platelet-lymphocytes ratio, monocyte-lymphocyte ratio (MLR), NLR, and systemic immune-inflammation index (SII), are also independent prognostic biomarkers in localized BC, in the neo-adjuvant and adjuvant setting, as well as in metastatic BC.^14,15^ In a previous study, in a cohort of breast cancer undergoing upfront surgery, preoperative systemic inflammatory biomarkers were independent predictors of disease recurrence in ER^+^ HER2^−^.^16^

Therefore, these inflammation-based scores correlate with cancer progression, metastasis, and outcomes.^17^ Although several potential molecular and blood cell-based prognostic biomarkers have been described, their widespread application is curtailed by the need for successful independent external validation. In this study, we aimed to establish and validate a prognostic model using an approach to predict DFS based on readily available baseline clinical-pathological prognostic factors and preoperative blood count-derived lymphocyte ratios.

## Materials and Methods

### Patient Population

#### Training Cohort

We retrospectively collected data from 710 patients diagnosed with BC at the Instituto do Cancer do Estado de São Paulo (ICESP; Sao Paulo, SP, Brazil) between January 2008 and December 2013. Eligible patients, defined as those with biopsy-proven breast cancer, who underwent surgery with curative intent, had complete information about clinical-pathological characteristics and follow-up data available. We excluded those patients previously diagnosed with other malignant neoplasia or who received previous anticancer treatment. Demographic and clinical-pathological variables at baseline (age, Eastern Cooperative Oncology Group - performance status (ECOG PS), tumor histology, blood cell counts and ratios, specifically, neutrophils, lymphocytes, monocytes, platelets, NLR, platelet to lymphocyte ratio, monocyte to lymphocyte ratio, systemic immune-inflammation (platelet count × NLR), and survival data were retrieved from electronic medical records. Only preoperative blood count results (counts taken as part of preoperative assessment) before systemic therapy/radiotherapy were considered in this study. The local ethics committee approved the study (EC. N^o^ 2.646.926). A waiver for the written informed consent was granted. The study was conducted in accordance with the Declaration of Helsinki.

### External Validation Cohorts (Cohorts V1 and V2)

Two independent early breast cancer cohorts served as external validation cohorts. Cohort V1 comprised 980 Indonesian patients diagnosed between January 2014 and December 2018. Cohort V2 included a dataset of 157 patients from Italy diagnosed between January 2008 and December 2014. All patients in these 2 external cohorts met the same eligibility criteria used in the training cohort, as stated above, and data regarding the same variables of interest were collected from these patients’ medical records. The study was performed according to approved protocols from each participating institution. A waiver for the written informed consent was granted from both institutions.

### Statistical Analysis

Descriptive statistics were used to summarize the data collected. Quantitative variables were reported as the median and interquartile range (IQR), while categorical variables were evaluated as absolute counts and frequencies.

Disease-free survival (DFS) was defined as the interval between the date of surgery and the date of the first relapse (both local and distant) or death, whichever occurs first; for patients alive and without recurrences, DFS was censored at the last available follow-up. Survival time was estimated according to the Kaplan-Meier method. The log-rank test assessed survival differences between subgroups of interest. A proportional hazard model was used to estimate Hazard Ratios (HR) and their 95% confidence intervals (95% CI). A multivariable analysis was performed and integrated factors that showed a *P*-value < .10 in univariate analysis. A stepwise forward procedure was implemented to identify independent factors associated with DFS. With this procedure, the most significant factor enters the model that contains no variables at the first step, then significance of the remaining factors is tested again and the most significant variable among the remaining enters the model; this procedure is performed in an iterative way and stops when there is no more significant factors outside of the model (ie, all remaining variables has a *P* > .05). During this iterative path, if a variable already in the model reaches a *P*-value > .10, the term is removed.

To reduce the influence of extreme values, inflammatory parameters were dichotomized according to a cutoff, which maximized the difference between DFS groups measured by the log-rank test.

A prognostic score was calculated based on variables independently associated with DFS at multivariable analysis in the training cohort. It was then applied to the 2 external validation cohorts by multiplying each beta coefficient by its corresponding covariate and summing the results. The prognostic score was then divided into 3 levels, the first group corresponded to the first quartile, the second level corresponded to values between the first and the third quartile, and the last level corresponded to values greater than the third quartile. The prognostic ability of the score was evaluated by calculating the Area Under Curve (AUC) of the ROC analysis. All analyses were performed with the IBM-SPSS Statistics for Windows, v.28.0, Armonk, NY: IBM Corp.

## Results

### Patient Characteristics

The baseline characteristics of all patients from the 3 cohorts (*N* = 1847) are summarized in Table 1. The training cohort comprised 710 patients with a median age at diagnosis of 60 years (IQR: 50-70 years). Mastectomy was performed in 396 (55.8%) and quadrantectomy in 314 (44.2%) patients. Most cases were pT1 stage (45.5%), and node-negative (58.2%) tumors. Regarding the molecular subtypes, tumors were luminal A and luminal B predominantly (71.4%), followed by HER2+ and triple-negative breast cancer (TNBC) (5.8% and 9.4%, respectively). In the Brazilian (training cohort) cohort, the median values of the NLR, PLR, MLR, and SII counts were 1.8, 118.6, 4.0, and 441, respectively.

**Table 1.** Characteristics of patients in the training cohort and in the 2 validation cohorts.

Variables		Training cohort (Brazil)	External validation cohort 1 (Indonesia)	External validation cohort 2 (Italy)
		(*n* = 710)	(*n* = 980)	(*n* = 157)^*^
		*n* (%)	*n* (%)	*n* (%)
Age	years, (interquartile range)	60 (50-70)	51 (44-58)	56 (48-63)
BMI	Normal	231 (32.5)	427 (43.6)	—
	Obese	231 (32.5)	99 (10.1)	—
	Overweight	232 (32.7)	236 (24.1)	—
	Underweight	13 (1.8)	218 (22.1)	—
	Missing	3 (0.4)	0	—
ECOG	0	435 (61.3)	559 (57.0)	151 (96.2)
	1+	271 (38.2)	421 (43.0)	6 (3.8)
	Missing	4 (0.5)	0	0
Surgery	Quadrantectomy	314 (44.2)	110 (11.2)	92 (58.6)
	Mastectomy	396 (55.8)	870 (88.8)	65 (41.4)
T	T1	323 (45.5)	21 (2.1)	108 (68.8)
	T2	322 (45.4)	222 (22.7)	44 (28.0)
	T3 + T4 Missing	64 (9.0) 1 (0.1)	737 (75.2) 0	5 (3.2) 0
N	Neg	413 (58.2)	280 (28.6)	83 (52.9)
	Pos Missing	291 (41.0) 6 (0.8)	700 (71.4) 0	74 (47.1) 0
Histological grade	G1 + G2	498 (70.1)	196 (20.0)	64 (40.7)
	G3	212 (29.9)	322 (32.9)	92 (58.6)
	Missing	0	462 (47.1)	1 (0.6)
Ki67	≤20%	409 (57.6)	558 (56.9)	63 (40.1)
	>20% Missing	250 (35.2) 51 (7.2)	421 (43.0) 1 (0.1)	49 (31.2) 45 (28.7)
Molecular subtype	Lum A + LumB^1^	507 (71.4)	563 (57.4)	79 (50.3)
	HER2	41 (5.8)	159 (16.2)	44 (28.0)
	TNBC	67 (9.4)	258 (26.3)	17 (10.8)
	Luminal Hybrid Missing	56 (7.9) 39 (5.5)	0 0	5 (3.2) 12 (7.7)
NLR	Median (IQR)	1.8 (1.4-2.4)	2.4 (1.8-3.3)	1.7 (1.3-2.3)
PLR	Median (IQR)	118.6 (92.9-147.5)	148.5 (113.4-195.6)	110.8 (91.6-140.7)
MLR	Median (IQR)	4.0 (3.2-5.0)	2.9 (2.0-3.8)	4.1 (3.3-5.1)
SII	Median (IQR)	441.1 (321.4-633.8)	663.9 (463.4-1001.6)	418.6 (276.5-561.3)

^*^Data regarding BMI was not collected in this cohort.

^1^Luminal A: ER+/HER2 − or PR+/HER2−; Luminal B: ER+/HER2 + or PR+/HER2+.

Abbreviations: BMI: body mass index; ECOG PS: Eastern Cooperative Oncology Group performance status; ER: estrogen receptor; LMR: monocyte to lymphocyte ratio; NLR: neutrophil to lymphocyte ratio; PLR: platelet to lymphocyte ratio; PgR: progesterone receptor; SII: systemic immune inflammation index; TNBC: triple negative breast cancer (ER−/PR−/HER2−).

The validation cohorts showed distinct clinicopathological characteristics compared with the training cohort. In the Asian cohort, most cases were clinically T2 + T3-stage at diagnosis, grade III (32.9%), and had lymph node involvement (N positive, 71.4%). Luminal A and luminal B subtypes accounted for the highest proportion (57.4%) of cases, followed by TNBC (26.3%) and HER2+ (16.2%), respectively. In addition, systemic inflammatory indexes were higher than observed in the Brazilian cohort. In the Italian cohort, the mean age of patients was 56 years and 58.6% underwent quadrantectomy. Most cases (68.8%) were clinically T1-stage at diagnosis and had no lymph node involvement (N negative, 52.9%).

Supplementary Table S1 provides a summary of the differences between the 3 cohorts. The majority of patients had ECOG PS score of 0 (61.3% in Brazilian cohort and 57% in Asian cohort, respectively). In Asian cohort, 88.8% patients underwent surgical mastectomy, 75.2% had T3-T4 stage at diagnosis, and 71.4% had lymph node involvement. There were significant differences between the training and validation cohorts in almost all of the presented features.

### Association Between the Inflammatory Indexes and Prognosis

**Figure 1.**
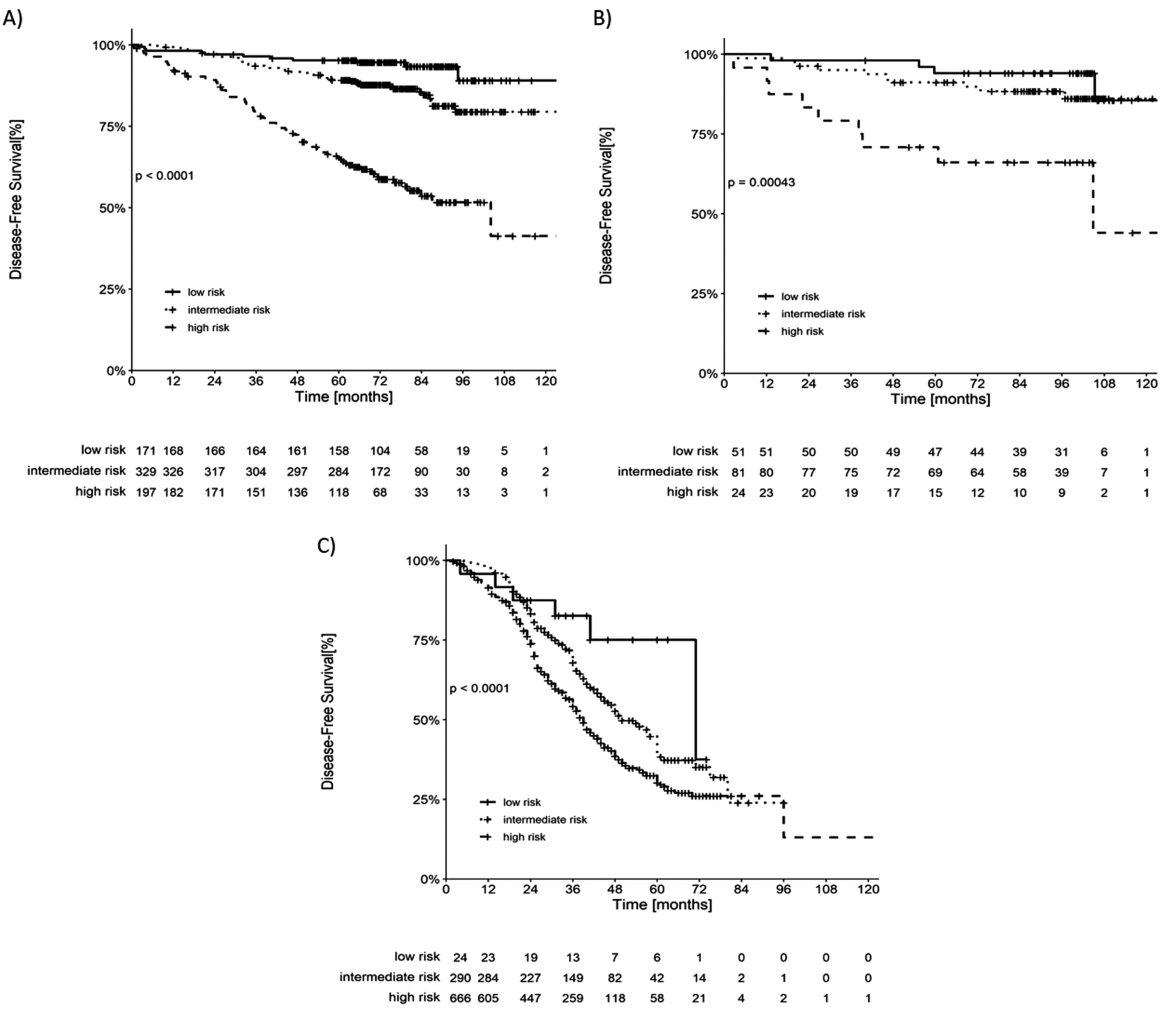
Kaplan-Meier plots of DFS according to baseline NLR. (**A**) DFS according to baseline NLR in the Brazilian cohort. (**B**) DFS according to baseline NLR in Italian cohort. (**C**) DFS according to baseline NLR in Indonesian cohort. Abbreviations: DFS: disease-free survival; NLR: neutrophil-to-lymphocyte ratio.

Median follow-up was 73 months in the training cohort, 31 months in the Asian cohort, and 99 months in the Italian cohort. Disease-free survival curves are shown in Fig. 1.

We evaluated the association between 4 inflammatory indexes (NLR, SII, MLR, and PLR) and prognosis. As a first step, each marker’s cutoff value was determined to maximize the HR for DFS based on the log-rank test. The best cutoff was found for NLR (1.35; *P* = .001), MLR (3.25; *P* = .001), and SII (250; *P* = .025).

### Identification of Independent Prognostic Variables in the Training Cohort

Univariable and multivariable analyses were performed in the training cohort. Univariable Cox proportional hazards analysis identified age, comorbidities, surgical modality, T stage, N stage, histological grade, ECOG, ER status, PgR status, molecular subtype, use of hormone therapy, systemic index, platelet to lymphocyte ratio, and monocyte to lymphocyte ratio as prognostic markers for DFS in patients undergoing surgery, these factors were entered into the multivariable analysis (Table 2).

**Table 2.** Univariable and multivariable Cox-regression analyses for disease-free survival in patients with early breast cancer in the training cohort.

Variable		Univariable		Multivariable	
		HR (95% CI)	*P*-value	HR (95% CI)	*P*-value
Age	(Continuous variable)	1.02 (1.00-1.03)	.008	--	
ECOG PS	0	1.00	<.0001	1.00	<.0001
	1	2.06 (1.40-3.02)		1.98 (1.32-2.98)	
	≥2	4.28 (2.82-6.51)		3.17 (1.98-5.07)	
BMI (kg/m^2^)	<24.9	1.00	.79		
	25.0-29.9	0.92 (0.62-1.37)			
	≥30	0.87 (0.58-1.31)			
Comorbidities	No	1.00	<.0001	1.00	.001
	Yes	2.36 (1.62-3.43)		1.98 (1.30-3.02)	
Surgery	Quadrantectomy/Segmentectomy	1.00	.022		
	Mastectomy	1.49 (1.06-2.11)			
T-stage	1	1.00	<.0001	1.00	.02
	2	1.73 (1.20-2.50)		1.37 (0.90-2.07)	
	3-4	2.88 (1.73-4.79)		2.11 (1.22-3.66)	
N-stage	Negative	1.00	<.0001	1.00	<.0001
	Positive	2.10 (1.51-2.94)		2.01 (1.39-2.89)	
Histological grade	G1	1.00	<.0001		
	G2	0.92 (0.58-1.47)			
	G3	1.89 (1.20-2.98)			
ER	Negative	1.00	.001		
	Positive	0.53 (0.36-0.77)			
PgR	Negative	1.00	<.0001	1.00	.001
	Positive	0.51 (0.36-0.71)		0.54 (0.37-0.78)	
HER2	Negative	1.00	.31		
	Positive	0.77 (0.46-1.28)			
Molecular subtype	LUMA + LUMB + Hybrid	1.00	.001		
	HER2	1.56 (0.83-2.91)			
	TNBC	2.31 (1.47-3.65)			
Chemotherapy	No	1.00	.21		
	Yes	1.26 (0.87-1.82)			
Radiotherapy	No	1.00	.98		
	Yes	1.01 (0.71-1.43)			
Hormonetherapy	No	1.00	<.0001		
	Yes	0.48 (0.34-0.69)			
NLR	<1.35 ≥1.35	1.00 2.37 (1.43-3.94)	.001	1.00 2.21 (1.28-3.81)	.004
MLR	<3.25 ≥3.25	1.00 0.57 (0.41-0.81)	.001		
SII	<250 ≥250	1.00 2.17 (1.10-4.26)	.025		

^*^ *P*-values were calculated using Wald chi-square tests; the *P*-values are 2-sided. Only variables that contributed statistically significantly to final models are included in the table.

Abbreviations: BMI: body mass index; ECOG PS: Eastern Cooperative Oncology Group performance status; ER: estrogen receptor; LMR: monocyte to lymphocyte ratio; NLR: neutrophil to lymphocyte ratio; PLR: platelet to lymphocyte ratio; PgR: progesterone receptor; SII: systemic immune inflammation index.

Patient and disease variables statistically significantly associated with lower survival in multivariable modeling included comorbidities (HR = 1.98; 95% CI, 1.30-3.02, *P* = .001), ECOG (HR = 3.17, 95% CI, 1.98-5.07, *P* < .0001), T stage (HR: 2.11, 95% CI, 1.22-3.66, *P* = .02), N stage (HR: 2.01, 95% CI, 1.39-2.89, *P* < .0001). Among the baseline laboratory markers considered, higher NLR was associated with lower DFS (HR: 2.21, 95% CI, 1.28-3.81, *P* = .04). Molecular subtype, chemotherapy, radiotherapy, hormonetherapy, and baseline monocyte to lymphocyte ratio and systemic immune-inflammation were not associated with DFS after adjustment for other factors.

### Development of a Prognostic Model for Early Breast Cancer

We built a prognostic model based on the variables selected in multivariable analysis for DFS in the training cohort (ECOG PS, T stage, N stage, progesterone receptor expression, presence of comorbidity, and NLR). We multiplied each factor by its beta coefficient estimated by the model and then summed up these values to calculate a score for each patient. The score was, therefore, calculated as follows: score = 0.68*(ECOG PS = 1) + 1.15*(ECOG PS ≥ 2) + 0.69*(comorbidities = yes) + 0.31*(Tstage = 2) + 0.75*(Tstage = 3-4) + 0.70*(Nstage = positive) + 0.79*(NLR ≥ 1.35 + 0.62*(PgRstatus = negative). The prognostic score was then divided into 3 groups based on the first and third quartile, these 2 quartiles being 1.30 and 2.26, respectively: patients with scores lower than the first quartile were classified in the low-risk group, those with a score higher than the third quartile in the high-risk group and the remaining patients in the intermediate-risk group (Table 3).

**Table 3.** DFS in the 3 different cohorts according to our prognostic model

Prognostic group	5 years DFS		
	Training cohort (Brazil)	External cohort 1 (Indonesia)	External cohort 2 (Italy)
Low	95%	75%	94%
Intermediate	89%	38%	91%
High	65%	30%	66%

The AUC of the ROC analysis was 0.74 (95% CI, 0.70-0.79), showing good prognostic ability in differentiating patients with or without DFS events.

## Discussion

In this study, we developed a prognostic model for DFS based on ECOG-PS, presence of comorbidities, T and N stage, PgR status, and NLR that was able to divide patients into 3 different risk groups for disease relapse, and then validated the model in 2 independent cohorts. Given that age is one of the strongest risk factors for breast cancer, the discriminatory performance of a model can be driven largely by variability in participant age. However, in our model after adjustment, age was eliminated as an independent predictor of DFS.

ECOG PS and blood-based biomarkers are known independent prognostic variables identified by standard Cox models in available datasets of patients with breast cancer, and our approach also validated these parameters. Despite poor prognostic characteristics, such as poor ECOG status and laboratory evidence of systemic inflammation, a relevant proportion of patients can still achieve long-term survival thanks to systemic treatment.^18,19^ This highlights the importance of considering prognostic biomarkers beyond the tumor environment.

Although other groups have derived prognostic models in a similar treatment setting, our work adds value by providing external validation in 2 patient cohorts (Asian and Italian). In Asia, there are wide variations in cancer incidence and mortality due to the different ethnic groups and socioeconomic status.^20^ Compared to the Italian group, most women in the Indonesian cohort had shorter follow-up, larger tumors and positive axillary lymph nodes. In contrast to a previous report showing that not only NLR, but also other biomarkers of systemic inflammation (eg, platelet to lymphocyte ratio) were associated with worse DFS,^21^ we did not find this in our study. Furthermore, baseline clinicopathological features were not related to NLR; therefore, it is unknown how imbalances in these inflammatory indices and other contributors might impact outcomes.

Including NLR substantially improved prognostic estimates in patients with BC undergoing surgical resection. The biological reasons why the NLR may be related to prognosis are multifactorial. NLR reflects a broad interaction between systemic inflammation and overall immune function and may serve as a proxy for the equilibrium between tumor-mediated inflammation and antitumor immunity.^22^ In multiple cancers, neutrophils accumulate in peripheral blood, and a high NLR has been positively associated with neutrophil infiltration into tumors^23^ and worse immune status.^24^

A lower circulating lymphocyte count may correlates with a reduced anti-tumor T-cell response, and tumor-infiltrating lymphocytes.^25^ The relationship between NLR and outcome in patients with cancer is probably a multifactorial process. While lymphocytes are cytotoxic to cancer cells, neutrophils are known to have a positive impact on cancer progression.^26^ Moreover, platelets interact with tumor cells, promoting invasion and angiogenic signaling.^27^ The PLR, in turn, has been established as prognostic biomarker for all molecular subtypes of breast cancer.^18,28^

White cell counts and their combinations (eg, NLR, SII, and MLR) have been highlighted because hematological tests are routinely performed for patients with cancer in clinical practice, and the activation of systemic inflammation is associated with changes in circulating white blood cells, such as neutrophilia and lymphocytopenia.^9^ In addition, lymphocytopenia has been associated with poor survival in many types of cancer, as tumors may induce lymphocyte apoptosis within the tumor microenvironment and in the peripheral circulation to avoid immune recognition.^29^

In line with our results, the prognostic value of the NLR was demonstrated by the Royal Marsden Hospital + neutrophil−lymphocyte ratio50 and the Gustave Roussy Score, improving the discriminatory ability for overall survival of patients with solid tumors.^30,31^ However, to what extent NLR values, as assessed by peripheral blood, reflect the effects of lymphocytes and neutrophils in local tumor-immunity remains unknown. Importantly, we validated our model in different cohorts, adding to the generalizability of the model, highlighting key clinical and pathological factors associated with prognosis and their relative contributions.

Our model performed well in these validation cohorts despite differing baseline patient characteristics and outcomes between Brazilian and external cohorts, which may reflect referral bias and varying treatment patterns. Notably, the NLR is a helpful and available prognostic marker but should be considered in combination with other patient clinical factors. The ability to more accurately predict individual outcomes is a key factor in personalizing therapy for breast cancer.

This study is likely to be representative of the population of women with breast cancer. Limitations of our study include patient heterogeneity, the Indonesian cohort differed greatly from the Brazilian and Italian cohorts, and this possibly introduced bias. Second, this is a retrospective study, and the model should be validated in a prospective cohort. Finally, NLR can be influenced by many factors, such as infections, treatment with steroids, or other stress triggers, which were not assessed in this setting. Exploring other predictive factors may help to better stratify adjuvant patient selection.

## Conclusion

In conclusion, we developed and externally validated a prognostic model based on readily available clinical and hematological variables to estimate DFS in patients with early breast cancer. Prospective validation is required to assess its discriminative performance in selected patient subgroups.

## Supplementary Material

Supplementary material is available at *The Oncologist* online.

oyad303_suppl_Supplementary_Tables_S1

## Data Availability

The raw data supporting the conclusions of this article have been deposited at Zenodo database and are available at: https://zenodo.org/record/7766970#.ZCFvEHZBxD9. Further information is available from the corresponding author upon reasonable request.
