# Supplementary material for: Development of a Prognostic Model for Early Breast Cancer Integrating Neutrophil to Lymphocyte Ratio and Clinical-Pathological Characteristics
Source: Oncologist. 2023 Nov 16;29(4):e447–54. doi: 10.1093/oncolo/oyad303 (PMC10994264; doi:10.1093/oncolo/oyad303)
Supplement: oyad303_suppl_Supplementary_Tables_S1 [file oyad303_suppl_supplementary_tables_s1.docx]

**Supplementary Material**

**Table S1. Comparison of clinicopathologic characteristics between training cohort and in the two validation cohorts**

| **Variables** | | **Training cohort** | **External validation cohort 1** | **p** | **External validation cohort 2** | **p** |
| --- | --- | --- | --- | --- | --- | --- |
|  |  | **(n=710)** | **(n=980)** |  | **(n=157)*** |  |
|  |  | n (%) | n (% ) |  | n (%) |  |
| **Age** | years, (interquartile range) | 60 (50-70) | 51 (44-58) | <0.0001 | 56 (48-63) | 0.15 |
| **BMI** | Normal | 231 (32.5) | 427 (43.6) | <0.0001 | - |  |
|  | Obese | 231 (32.5) | 99 (10.1) |  | - |  |
|  | Overweight | 232 (32.7) | 236 (24.1) |  | - |  |
|  | Underweight | 13 (1.8) | 218 (22.1) |  | - |  |
|  | Missing | 3 (0.4) | 0 |  | - |  |
| **ECOG** | 0 | 435 (61.3) | 559 (57) | 0.06 | 151 (96.2) | <0.0001 |
|  | 1+ | 271 (38.2) | 421 (43) |  | 6 (3.8) |  |
|  | Missing | 4 (0.5) | 0 |  | 0 |  |
| **Surgery** | Quadrantectomy | 314 (44.2) | 110 (11.2) | <0.0001 | 92 (58.6) | 0.001 |
|  | Mastectomy | 396 (55.8) | 870 (88.8) |  | 65 (41.4) |  |
| **T-stage** | T1 | 323 (45.5) | 21 (2.1) | <0.0001 | 108 (68.8) | <0.0001 |
|  | T2 | 322 (45.4) | 222 (22.7) |  | 44 (28.0) |  |
|  | T3+T4 | 64 (9.0) | 737 (75.2) |  | 5 (3.2) |  |
|  | Missing | 1 (0.1) | 0 |  | 0 |  |
| **N-stage** | Neg | 413 (58.2) | 280 (28.6) | <0.0001 | 83 (52.9) | 0.48 |
|  | Pos | 291 (41.0) | 700 (71.4) |  | 74 (47.1) |  |
|  | Missing | 6 (0.8) | 0 |  | 0 |  |
| **Histological grade** | G1+G2 | 498 (70.1) | 196 (20) | <0.0001 | 64 (40.7) | <0.0001 |
|  | G3 | 212 (29.9) | 322 (32.9) |  | 92 (58.6) |  |
|  | Missing | 0 | 462 (47.1) |  | 1 (0.6) |  |
| **Ki67** | ≤20% | 409 (57.6) | 558 (56.9) | 0.04 | 63 (40.1) | 0.24 |
|  | >20% | 250 (35.2) | 421 (43.0) |  | 49 (31.2) |  |
|  | Missing | 51 (7.2) | 1 (0.1) |  | 45 (28.7) |  |
| **Molecular subtype** | Lum A +LumB | 507 (71.4) | 563 (57.4) | <0.0001 | 79 (50.3) | <0.0001 |
|  | HER2 | 41 (5.8) | 159 (16.2) |  | 44 (28) |  |
|  | TNBC | 67 (9.4) | 258 (26.3) |  | 17 (10.8) |  |
|  | Luminal Hybrid | 56 (7.9) | 0 |  | 5 (3.2) |  |
|  | Missing | 39 (5.5) | 0 |  | 12 (7.7) |  |
| **NLR** | Median (IQR) | 1.8 (1.4-2.4) | 2.4 (1.8-3.3) | <0.0001 | 1.7 (1.3-2.3) | 0.06 |
| **PLR** | Median (IQR) | 118.6 (92.9-147.5) | 148.5 (113.4-195.6) | <0.0001 | 110.8 (91.6-140.7) | 0.15 |
| **MLR** | Median (IQR) | 4.0 (3.2-5.0) | 2.9 (2.0-3.8) | <0.0001 | 4.1 (3.3-5.1) | 0.23 |
| **SII** | Median (IQR) | 441.1 (321.4-633.8) | 663.9 (463.4-1001.6) | <0.0001 | 418.6 (276.5-561.3) | 0.04 |
